# Quality of routine facility data for monitoring priority maternal and newborn indicators in DHIS2: A case study from Gombe State, Nigeria

**DOI:** 10.1371/journal.pone.0211265

**Published:** 2019-01-25

**Authors:** Antoinette Alas Bhattacharya, Nasir Umar, Ahmed Audu, Habila Felix, Elizabeth Allen, Joanna R. M. Schellenberg, Tanya Marchant

**Affiliations:** 1 Faculty of Infectious and Tropical Diseases, London School of Hygiene & Tropical Medicine, London, United Kingdom; 2 State Primary Health Care Development Agency, Gombe, Nigeria; 3 Faculty of Epidemiology and Population Health, London School of Hygiene & Tropical Medicine, London, United Kingdom; Tulane University School of Public Health and Tropical Medicine, UNITED STATES

## Abstract

**Introduction:**

Routine health information systems are critical for monitoring service delivery. District Heath Information System, version 2 (DHIS2) is an open source software platform used in more than 60 countries, on which global initiatives increasingly rely for such monitoring. We used facility-reported data in DHIS2 for Gombe State, north-eastern Nigeria, to present a case study of data quality to monitor priority maternal and neonatal health indicators.

**Methods:**

For all health facilities in DHIS2 offering antenatal and postnatal care services (n = 497) and labor and delivery services (n = 486), we assessed the quality of data for July 2016-June 2017 according to the World Health Organization data quality review guidance. Using data from DHIS2 as well as external facility-level and population-level household surveys, we reviewed three data quality dimensions—completeness and timeliness, internal consistency, and external consistency—and considered the opportunities for improvement.

**Results:**

Of 14 priority maternal and neonatal health indicators that could be tracked through facility-based data, 12 were included in Gombe’s DHIS2. During July 2016-June 2017, facility-reported data in DHIS2 were incomplete at least 40% of the time, under-reported 10%-60% of the events documented in facility registers, and showed inconsistencies over time, between related indicators, and with an external data source. The best quality data elements were those that aligned with Gombe’s health program priorities, particularly older health programs, and those that reflected contact indicators rather than indicators related to the provision of commodities or content of care.

**Conclusion:**

This case study from Gombe State, Nigeria, demonstrates the high potential for effective monitoring of maternal and neonatal health using DHIS2. However, coordinated action at multiple levels of the health system is needed to maximize reporting of existing data; rationalize data flow; routinize data quality review, feedback, and supervision; and ensure ongoing maintenance of DHIS2.

## Introduction

Routine health information systems are critical for monitoring service delivery. One distinctive feature of routine health information systems is the availability of data at a frequency and level of disaggregation seldom possible through nationally representative household surveys such as the Demographic and Health Surveys and Multiple Indicator Cluster Surveys. [1–3]

Global initiatives including the Sustainable Development Goals and Countdown to 2030 emphasize the contribution of routine health information systems to monitor progress and enable course correction. [4–6] Two major maternal and newborn health initiatives, Ending Preventable Maternal Mortality and Every Newborn Action Plan, have identified strategies to achieve goals for reduced maternal and newborn mortality by 2030 to a global average of 70 per 100,000 live births and 12 per 1,000 live births, respectively. Both initiatives have identified priority indicators as signals for progress, with a vision that facility-based data should contribute to monitoring. [7, 8] The District Health Information System, version 2 (DHIS2), is a flexible open source electronic information system currently used in over 60 countries to manage and visualize routine health data, particularly facility-based data.[9] Here, we present a case study for Gombe State, north-eastern Nigeria, to examine the availability and quality of routine facility data in DHIS2 for this monitoring purpose.

A routine health information system is a sub-system of a national health information system’s effort to capture, process, report, and use information to support policymaking and program implementation. [10, 11] A facility-based information system is a further sub-system that includes data captured by health facility workers during their day-to-day activities. These facility-based data include paper-based and electronic-based medical records, service delivery registers, and aggregate service delivery reports. When facility-based data are of sufficient quality, they can be used at the facility level for effective clinical management, at the district-level to understand the extent to which their facilities are functioning as intended, and at the state- and national-levels to review policies and allocation of resources. [1, 12] At all levels of the health system, good quality facility-based data can contribute to reliable estimates of service delivery coverage to understand if communities are accessing and receiving needed services, such as the proportion of facility births attended by a skilled health worker. [1, 3, 12, 13].

While facility-based information systems are often unable to maintain the good quality needed for monitoring [14, 15], DHIS2 is considered an innovation for transmitting and aggregating data faster than paper-based information systems and for improving data quality by limiting errors in how data are transmitted and aggregated from the facility to higher levels of the health system. Further, DHIS2 has the potential to promote program monitoring because its digital platform increases the accessibility of data for health managers and stakeholders at the district-, state-, and national levels. [3, 9, 16]

With Nigeria having one of the highest maternal mortality ratios and newborn mortality rates in the world (576 maternal deaths per 100,000 live births in 2015 and 37 newborn deaths per 1,000 live births in 2017), the Government has developed action plans to reduce preventable deaths for mothers and newborns and has made considerable investment in strengthening information systems, including DHIS2, to support performance management and service delivery. [17–22]

The aim of this study was to determine the quality of routine facility-based data in DHIS2 to monitor priority maternal and neonatal health indicators in Gombe State, north-eastern Nigeria. Using the World Health Organization data quality review toolkit, we focused on metrics for the data quality dimensions of completeness and timeliness, internal consistency, and external consistency. [23] For data defined as poor quality by the toolkit, we discussed opportunities for improvement.

## Methods

### Ethical approval

Gombe State approval for the study was obtained from Gombe State Ministry of Health. Ethical approval was obtained from the London School of Hygiene & Tropical Medicine (reference 14091).

### Study setting

Gombe State has a projected population of 2.9 million (2006 census: 2.4 million) and is located within north-eastern Nigeria, where maternal and newborn mortality are estimated to be higher than the rest of the country (1,549 maternal deaths per 100,000 live births in 2015 and 35 neonatal deaths per 1,000 live births in 2017). [22, 24–26] In 2017, Gombe State had a total of 615 health facilities across 11 Local Government Areas (LGA, equivalent to a district); each LGA has 10–11 political wards (114 wards, total). As in other states in Nigeria, Gombe facility staff generally complete 13 paper-based registers to document the services they provide. Every month, a subset of data in these registers are tallied and summarized in a paper-based report and sent to the LGA (district) health office to be entered into DHIS2.

### Data sources

We accessed three data sources for this study: facility-reported data in DHIS2, an external facility survey, and an external household survey as described below.

In 2017, DHIS2 contained monthly reports for 615 Gombe public and private health facilities across 11 districts: 587 primary facilities offering basic preventative and curative services and 28 referral facilities offering specialized care. Of these, 471 of the 587 primary facilities had been appointed to provide antenatal care and postnatal care services, 460 of the 587 primary facilities provided labor and delivery services, and 26 of the 28 referral facilities were equipped to provide both types of services, in addition to specialized care. Therefore, in total, 497 facilities provided antenatal and postnatal care services and 486 facilities provided labor and delivery services. For these 497 and 486 facilities, respectively, monthly aggregated DHIS2 data for the reference year July 2016-June 2017 were downloaded at one time and included 15 maternal and newborn health-related data elements. Additionally, we downloaded data for July 2013-June 2016 as comparison years for assessing the consistency of data over time.

In July 2017, a facility-level survey was conducted in 97 primary and 18 referral facilities across Gombe to assess their readiness to provide maternal and newborn health services. Detailed methods are reported elsewhere.[27] Briefly, these primary and referral facilities were a state-wide random sample drawn from all government-owned primary health facilities and a census of all 18 government-owned referral health facilities. The facility survey protocol was similar to a Service Availability and Readiness Assessment, which included an inventory of equipment and supplies that were available and functioning on the day of survey; an inventory of staff employed at the facility, their cadre, training and whether they were present on the day of survey; and an interview with the in-charge of the facility about the services available at that facility and about recent supervision visits they had received. Additionally, this survey included data extraction from the facility’s paper-based antenatal and postnatal care register and the labor and delivery register (Nigeria health management information system, version 2013).[28] A trained third party data collection team tallied and recorded the register data for each month of the six-month period immediately prior to the survey: January-June 2017. We compared the facilities’ paper-based register data with the facilities’ data downloaded from DHIS2. These extracted data are shown in Table 1.

**Table 1:** Priority maternal and newborn health data in Gombe State’s facility registers and reports in DHIS2.

Priority maternal and newborn health data element:	Gombe’s routine health information system	
	Facility registers	DHIS2
**Main denominators**		
Facility deliveries	x	x
Facility live births	x	x
First antenatal care visits	x	x
**Coverage indicators: care for all women and newborns**		
Four or more antenatal care visits	x	x
Skilled attendant at birth	x	x
*Institutional delivery*		
Oxytocin immediately after birth for prevention of postpartum hemorrhage	x	
Early postpartum-postnatal care for woman and newborn*	x	x
*Met need for family planning*		
Essential newborn care	x	
Content of antenatal care		
Hypertension: blood pressure taken		
Anemia: blood test	x	x
Proteinuria: urine test	x	x
Iron supplementation	x	x
Tetanus protection	x	x
Intermittent preventive treatment of malaria in pregnancy	x	x
Known HIV status or tested for HIV and received results	x	x
Counseling on pregnancy complications		
Content of postnatal care		
No pre-lacteal feeds during first three days of life		
BCG vaccination during postnatal period	x	x
Polio vaccination at birth	x	x
*Respectful maternity care*		
Exclusive breastfeeding up to 6 months	x	x

Notes:

Indicators in *italic* type cannot be calculated only from routine facility data.

*Gombe facility registers and DHIS2 track early postpartum-postnatal care within 1 and 3 days of birth. To ensure exclusion of care provided to mothers and newborns during labor and delivery, we used early postpartum-postnatal care within 3 days of birth.

Also in July 2017, a household-level survey was conducted in catchment areas of the 97 primary facilities from the July 2017 facility survey to assess access to and quality of maternal and newborn services. [27] These catchment areas represented 79 enumeration areas: some facilities serving more than one enumeration area. All households in each enumeration area were surveyed (or in a segment of between 75 households from the enumeration area if more than 75 households were present). The household survey comprised of three modules. (1) A household module asked all household heads about characteristics of the household, ownership of commodities and registered all normally resident people in the household. (2) A women’s module asked all women aged 13–49 years and normally resident in the household about the health care available to them, their recent contact with frontline workers and their birth history in the two years preceding the survey. (3) A mother’s module asked all women who reported a birth in the last two years (identified in the women’s module) a detailed set of questions about their contact with health services across the continuum of care from pregnancy to postnatal care. Informed consent was obtained at the community leadership-level and at the individual-level for each respondent; all invited participants agreed be interviewed. Among 965 surveyed women who reported a live birth in the 12 months prior to the survey, 588 women had visited the facility at least once during their pregnancy and 377 women gave birth at a facility. For DHIS2 reported indicators that were also estimated in the household survey, we compared estimates from this household survey to those from the 79 matching facilities in DHIS2. Calculations of point estimates and their 95% confidence intervals were done using the svyset Stata command (StataCorp, College Station, USA) to adjust for clustering at the enumeration area-level.

### Selection of priority maternal and newborn health indicators to assess in Gombe’s DHIS2

To determine globally-defined priority maternal and newborn health data in DHIS2, we referred to the Ending Preventable Maternal Mortality and Every Newborn Action Plan strategy documents which described priority indicators to monitor progress towards targets during the Sustainable Development Goals era. [7, 8] For content of care indicators that were referenced by these strategy documents, but not yet fully defined, we referred to indicators defined in Carvajal-Aguirre et al. [29]

We focused our data quality review on health services that should be received by all pregnant women and newborns accessing either primary or referral health facilities. Therefore, rare events and outcomes such as deaths, adolescent births, pre-term births, deliveries by caesarean section, and kangaroo mother care were excluded from our analyses.

For Gombe State, we identified 14 priority maternal and newborn health indicators that were captured at the facility-level by health care workers. (Table 1) These 14 indicators are made up of 17 distinct data elements contained within the paper-based facility registers, including three denominators to determine how many women and newborns have accessed these facilities for services: women who visited the facility at least once during their pregnancy; women who gave birth in a facility; and live births among the facility births.

For Gombe State, 15 of these 17 distinct data elements were reported in DHIS2; the data for women receiving oxytocin for the prevention of postpartum hemorrhage and newborns receiving essential care were captured in facility registers, but not reported in DHIS2. Therefore, the final set of data assessed included 15 data elements used to calculate 12 priority indicators.

### Data quality assessment

We reviewed the quality of the DHIS2 data according to metrics of three routine data quality dimensions outlined by the World Health Organization data quality review toolkit: completeness and timeliness; internal consistency; and external consistency. [23] Table 2 outlines the data quality metrics assessed, the criterion for each metric, and the data sources used. A stratified analysis was performed by facility type for primary and referral facilities.

**Table 2:** Data quality metrics and data sources reviewed.

Data quality metric	Analysis/calculation, WHO guidance for quality*	Source(s)	Facilities
***Data quality dimension 1*: Completeness and timeliness of data**			
Completeness of facility reporting in DHIS2: Extent to which each facility submitted a monthly summary report	Number and % of facility’s expected monthly reports actually submitted. Completeness of facility reporting should be 75% or higher*	DHIS2	497 ANC-PNC facilities (471 primary, 26 referral facilities) 486 labor and delivery facilities (460 primary, 26 referral facilities)
Timeliness of facility reporting in DHIS2: Extent to which each facility submitted a monthly summary report on or before specified timeline	Number and % of facility’s expected monthly reports actually submitted on time. *No specified guidance on timeliness of facility reporting*	DHIS2	497 ANC-PNC facilities 486 labor and delivery facilities
Completeness of indicator data in DHIS2: Extent to which select indicator data within submitted reports contained a non-missing (or non-zero) value	Number and % non-missing values for a given indicator in expected monthly reports. Non-missing values for a given indicator should be present in 90% or more monthly reports*	DHIS2**	497 ANC-PNC facilities 486 labor and delivery facilities
***Data quality dimension 2*: Internal consistency of data**			
Consistency over time: Extent to which indicator data exhibit similar patterns as in previous seasons	Ratio of value of indicator for reference year to the mean of preceding 3 years *Ratio of value of indicator for reference year should be within* ±33% of mean of preceding 3 years*	DHIS2	497 ANC-PNC facilities 486 labor and delivery facilities
Outliers in reference year: Extent to which the values reported for a given indicator are extreme and potentially implausible	Number of moderate outliers (±2-3SD from the mean) and extreme outliers (±3SD from the mean) of monthly values during the reference year *Value of indicator should be within* *±2SD from the mean*	DHIS2	497 ANC-PNC facilities 486 labor and delivery facilities
Consistency between related data: Extent to which the values for two or more indicators exhibit the predicted relationship	Ratio for values of indicator-pairs that have a predictable relationship *Indicator-pairs that should be roughly equal should be within* *±10% of each other*	DHIS2	497 ANC-PNC facilities 486 labor and delivery facilities
Consistency between original facility registers and reported data in DHIS2: Extent to which values for given indicators agree between two internal data sources	Ratio of indicator values in original facility register count to facility monthly summary report in DHIS2 *Indicator values in original facility register count and facility monthly report in DHIS2 should be within* *±10% of each other*.	Facility registers; DHIS2	110 ANC-PNC facilities (92 primary, 18 referral) 108 labor and delivery facilities (90 primary, 18 referral)
***Data quality dimension 3*: *External consistency of data***			
Consistency between household surveys and reported data in DHIS2: Extent to which values for given indicators agree with an external data source	Ratio of indicator values in household surveys for facility catchment areas to matching facilities in DHIS2 *Indicator values from facility reports in DHIS2 should be within* *±33% of household survey value or within confidence limits of household survey*.	Household surveys; DHIS2	79 ANC-PNC facilities (primary facilities) 79 labor and delivery facilities (primary facilities, same facilities as ANC-PNC facilities)

Notes:

ANC = antenatal care, PNC = postnatal care, SD = standard deviation.

* WHO threshold for good data quality should be adapted for each health program and/or country.

**For the period under review, downloaded data from Gombe State’s DHIS2 did not distinguish between missing values and true zero values; both are presented as missing values.

## Results

We present the quality of 15 data elements which represented 12 priority maternal and neonatal health indicators included in DHIS2 for Gombe State.

### Completeness and timeliness of facility reporting

**Fig 1:**
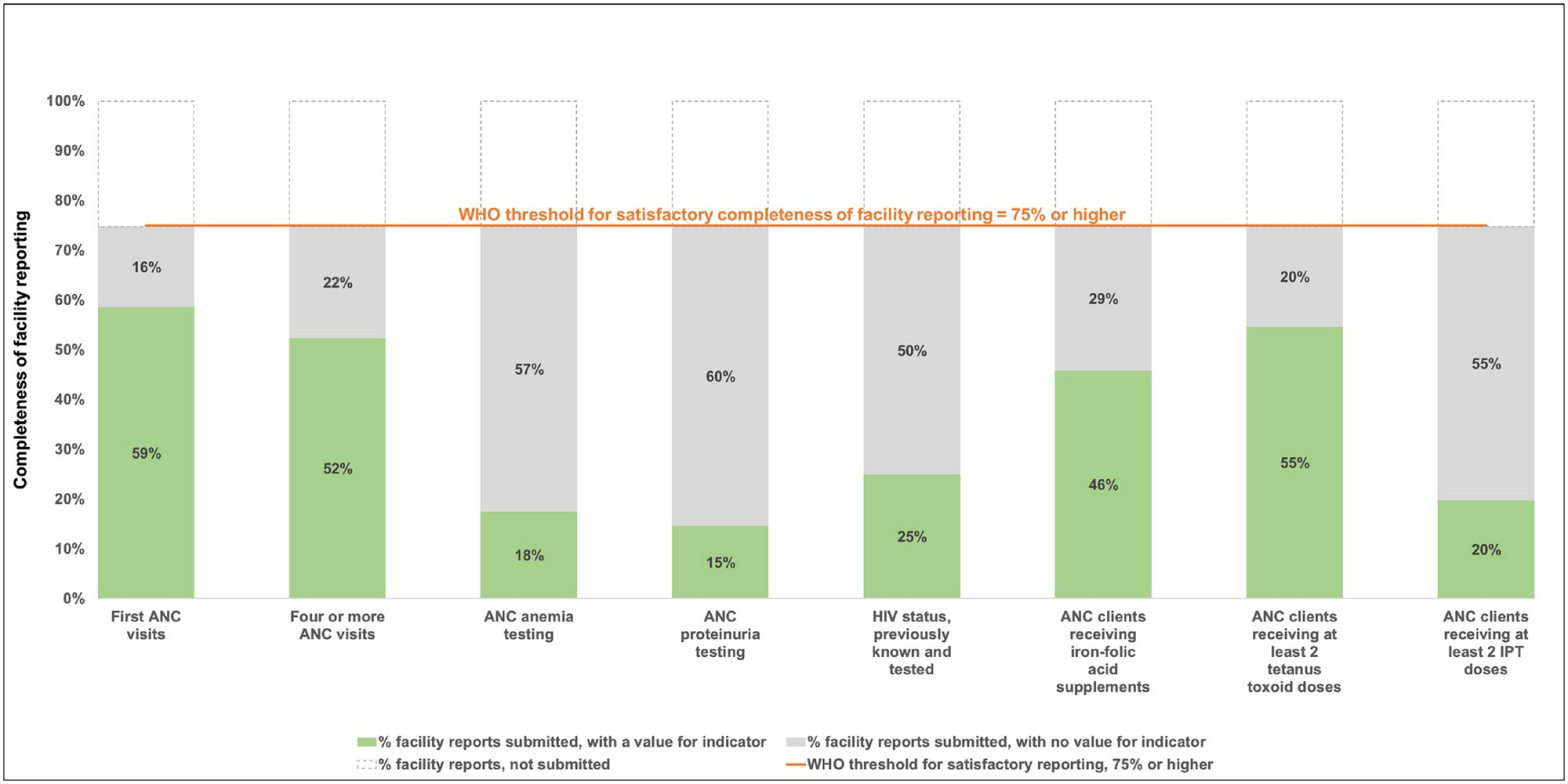
Antenatal care: Completeness of facility reporting and indicator data in Gombe State, Nigeria, July 2016-June 2017. Notes: ANC = antenatal care; HIV = human immunodeficiency virus; IPT = intermittent preventive treatment of malaria in pregnancy.

**Fig 2:**
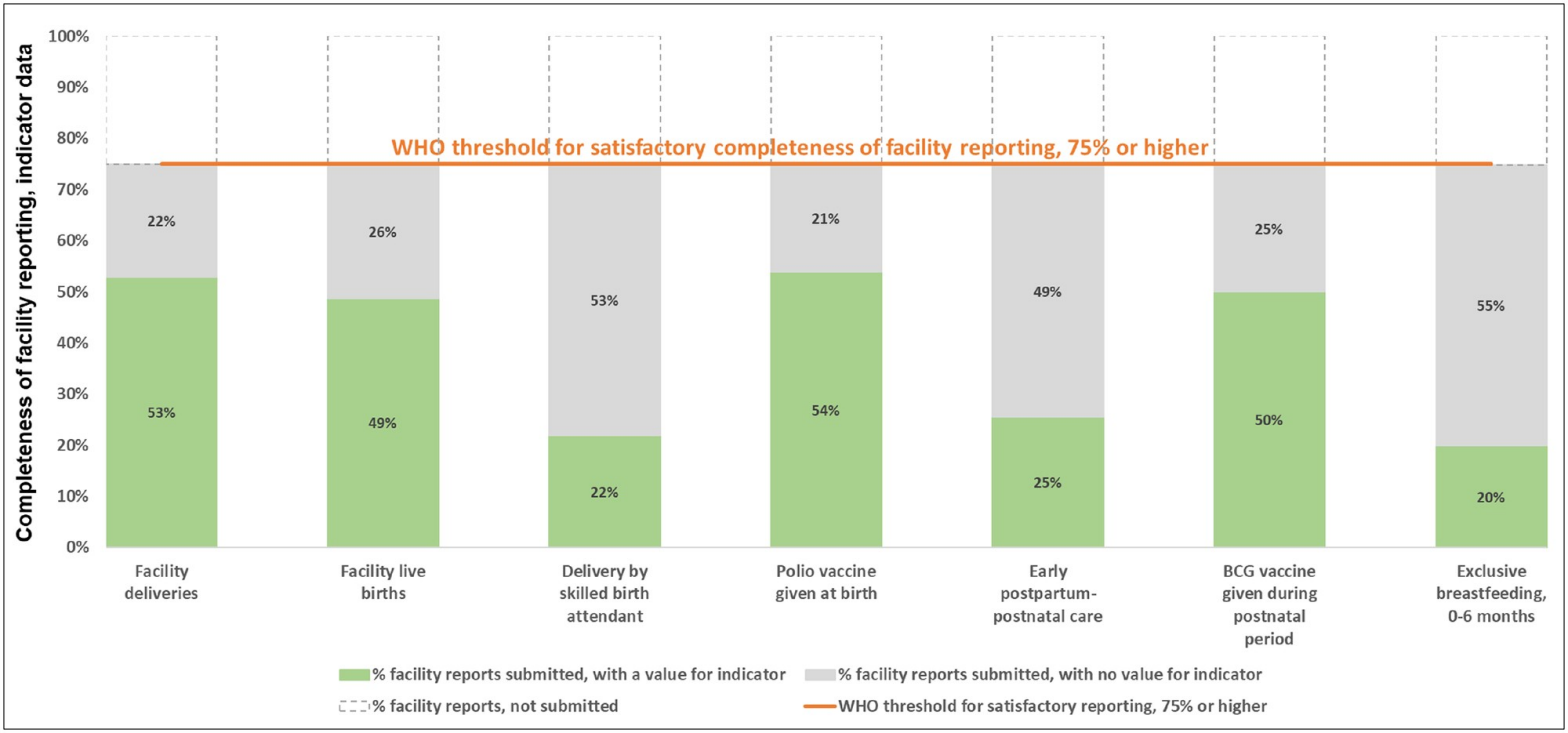
Labor, delivery and postnatal care: Completeness of facility reporting and indicator data in Gombe State, Nigeria, July 2016-June 2017.

For settings such as Gombe, the World Health Organization guidance defined 75% to represent satisfactory completeness of facility reporting, that is each facility annually submitted at least nine of the 12 expected reports. [23] In Gombe State, facilities providing antenatal and postnatal care services (n = 497 facilities) and labor and delivery services (n = 486 facilities) submitted, on average, 75% of the expected reports during July 2016-June 2017 (nine of 12 expected reports submitted per year, standard deviation: 2.9 reports). Of these, 84% of reports were submitted on-time, although referral facilities were less likely than primary facilities to submit their reports on time (p<0.01 for both antenatal and postnatal care facilities and labor and delivery facilities). Figs 1 and 2 present the completeness of facility reporting, alongside completeness of indicator data described below.

### Completeness of priority maternal and newborn data in DHIS2

To assess the completeness of indicator data (the extent to which health facilities reported for specific indicators), we observed that Gombe’s DHIS2 data did not distinguish between missing values and true zero values. For example, a remote facility may have been equipped to provide antenatal care services but had no clients for antenatal care during a review month (true zero value); in contrast, a facility may have provided antenatal care services to clients but did not include this in their monthly report (missing value). In Gombe’s DHIS2, both situations are presented as missing data.

The World Health Organization defined completeness of indicator data to be satisfactory when less than 10% of the expected data were missing values. In Figs 1 and 2, the priority data elements in DHIS2 with the least missing values were for the number of times pregnant women visited a facility for antenatal care (first antenatal care visits, four or more antenatal care visits), deliveries taking place in a facility (facility deliveries), the provision of tetanus toxoid vaccinations to pregnant women, and the provision of Bacillus Calmette-Guérin (BCG) vaccinations to newborns. Facilities reported a value for these data in at least 52% of the expected monthly reports and 65% of submitted reports. By contrast, the priority data elements with the most missing values were for the provision of screening tests for anemia and proteinuria as well as malaria intermittent preventive treatment. Facilities reported a value for these data in less than 25% of expected monthly reports and less than 33% of submitted monthly reports.

Differences in the completeness of indicator data were noted by facility type. Primary facilities were more likely than referral facilities to report that any woman and her newborn received early postpartum-postnatal care (early postpartum-postnatal care) (p<0.01), any newborn was given a polio vaccine at birth (p<0.01), and any mother reported exclusively breastfeeding her infant up to six months of age (p = 0.01). Referral facilities were more likely to report any pregnant women received a screening test for anemia (p = 0.03) and for proteinuria (p = 0.02).

### Consistency over time

When assessing the extent to which a data element’s reported value was consistent over time, the World Health Organization guidance recommended that the reported value for the reference year be within ±33% of the mean value for the preceding three years, taking into consideration any expected changes in the patterns of service delivery. For Gombe State, facilities were more likely to report consistently, compared to the preceding three years, for 7 of 15 data elements (Table 3): first antenatal care visits; four or more antenatal care visits; women receiving at least two doses of malaria intermittent preventive treatment during pregnancy; facility deliveries; deliveries by a skilled birth attendant; newborns receiving BCG vaccinations; and mothers reporting exclusive breastfeeding through six months of age. Facilities did not report consistently, compared to the preceding three years, for women having a live birth in a facility (live births); early postpartum-postnatal care; antenatal care commodities and services provided, except for the provision of malaria intermittent preventive therapy; and newborns receiving the polio vaccine at birth.

**Table 3:** Consistency over time for priority maternal and neonatal health indicators in DHIS2: Gombe State, Nigeria, July 2013-June 2017.

	DHIS2 count for priority maternal and newborn health indicators					
	Year 1, July 2013-June 2014	Year 2, July 2014-June 2015	Year 3, July 2015-June 2016	Year 4, July 2016-June 2017	Mean of Year 1-Year 3	Ratio of Year 4 to Mean of Year 1-Year 3
**Main denominators**						
Facility deliveries	52,055	53,330	83,785	65,312	63,057	1.04
Facility live births	31,214	25,089	50,049	51,001	35,451	***1*.*44***
First antenatal care visits	110,534	79,848	152,992	143,786	114,458	1.26
**Coverage: care for all women and newborns**						
Four or more antenatal care visits	63,642	53,026	104,344	96,185	73,671	1.31
Deliveries by a skilled birth attendant	11,059	22,573	17,404	20,205	17,012	1.19
Early postpartum-postnatal care for woman and newborn	2,738	2,906	6,675	7,067	4,106	***1*.*72***
Content of antenatal care:						
Anemia testing	36,269	35,216	63,076	65,737	44,854	***1*.*47***
Proteinuria testing	41,340	34,193	64,536	76,559	46,690	***1*.*64***
Known HIV status, previously known or tested	45,528	63,973	102,591	138,894	70,697	***1*.*96***
Iron-folic acid supplementation	106,955	123,200	209,338	347,828	146,498	***2*.*37***
At least 2 doses of TT	43,542	46,357	93,202	90,046	61,034	***1*.*48***
At least 2 doses of IPT	32,995	21,743	27,802	21,579	27,513	0.78
Content of postnatal care:						
BCG vaccine given during postnatal period	66,279	63,153	115,012	99,142	81,481	1.22
Polio vaccine given at birth	42,431	46,667	84,040	78,396	57,713	***1*.*36***
Exclusive breastfeeding up to 6 months of age	18,724	12,364	26,843	23,798	19,310	1.23

**Notes**:TT = tetanus toxoid; IPT = intermittent preventive treatment in pregnancy for malaria; BCG = Bacillus Calmette-Guérin. Values in ***italic and bold*** indicate data that were considered inconsistent over time. According to WHO guidance, ratios <0.67 or >1.33 indicate reported data in DHIS2 for reference year was inconsistent with the mean of the preceding 3 years.

We observed differences in consistency over time by facility type, with referral facilities more likely to report an inconsistent and higher number of events for July 2016-June 2017 compared to the mean of the previous three years for six data elements (for each data element: p<0.05): four or more antenatal care visits; facility deliveries; live births; deliveries by a skilled birth attendant; newborns receiving a polio vaccination; and newborns receiving a BCG vaccination.

### Outliers in the reference year

When assessing indicator data for unlikely or extreme values (outliers) in the reference year, the World Health Organization guidance defined an individual monthly value of a given data element to be a moderate outlier if it was between two and three standard deviations from the mean value and an extreme outlier if it was more than three standard deviations from the mean value for the year.

For Gombe State, outliers were present during the reference year for nine of the 15 data elements (Table 4). Primary facilities were responsible for reporting all outliers, with the monthly outlier values being higher than the reported mean number of events for the year. Primary facilities reported moderate monthly outlier values for: first antenatal care visits; facility deliveries; deliveries by a skilled birth attendant; newborns receiving a BCG vaccination; and mothers reporting exclusively breastfeeding their infant up to six months of age. Primary facilities reported extreme monthly outlier values for pregnant women receiving a screening test for proteinuria; women whose HIV status was known or tested for; pregnant women given iron-folic acid supplementation; and pregnant women receiving at least two doses for malaria intermittent preventive therapy. Three extreme outliers were reported in October 2016, mostly due to one primary health facility’s reported values contributing 60% towards Gombe State’s aggregate value for women receiving at least two doses of IPT, 87% towards the aggregate value for women who were tested for HIV or with previously known HIV status, and 90% the aggregate value for women who received iron-folic acid supplementation.

**Table 4:** Outliers for priority maternal and neonatal health indicators in DHIS2: Gombe State, Nigeria, July 2016-June 2017.

	Jul 2016	Aug 2016	Sep 2016	Oct 2016	Nov 2016	Dec 2016	Jan 2017	Feb 2017	Mar 2017	Apr 2017	May 2017	Jun 2017	Jul 2016- Jun 2017
**Main denominators**													
Facility deliveries	4,662	4,197	3,881	4,418	4,926	5,537	5,700	5,574	5,704	6,284	6,124	**8,305**	65,312
Facility live births	3,918	3,632	3,201	3,494	3,974	4,130	4,467	5,031	5,009	4,094	5,027	5,024	51,001
First antenatal care visits	10,815	12,053	10,148	12,646	13,863	12,197	**15,382**	11,365	12,282	10,358	12,895	9,782	143,786
**Coverage: care for all women and newborns**													
Four or more antenatal care visits	5,846	8,397	6,367	8,349	9,856	8,028	10,009	8,276	8,174	7,405	8,009	7,469	96,185
Deliveries by skilled birth attendant	1,122	868	758	1,439	1,434	1,603	1,780	1,538	2,287	1,650	1,380	**4,346**	20,205
Early postpartum-postnatal care	423	523	449	523	616	560	783	563	666	567	698	696	7,067
Content of antenatal care:													
Anemia testing	4,286	5,656	5,897	4,770	5,798	6,293	6,386	5,269	6,338	4,792	5,511	4,741	65,737
Proteinuria testing	4,353	4,379	5,858	4,684	5,341	6,361	7,189	5,447	***13*,*457***	6,262	7,409	5,819	76,559
Known HIV status, previously known or tested	7,301	7,476	7,744	***55*,*355***	8,087	7,429	8,146	7,103	8,477	7,550	7,833	6,393	138,894
Iron-folic acid supplementation	14,316	14,533	13,905	***148*,*304***	17,954	15,628	23,231	18,108	28,338	16,963	19,327	17,221	347,828
At least 2 doses of TT	6,079	6,895	5,830	8,666	8,510	7,312	8,591	6,936	7,233	7,214	8,799	7,981	90,046
At least 2 doses of IPT	1,749	1,610	1,435	***3*,*896***	2,001	1,480	1,799	1,407	1,472	1,417	1,635	1,678	21,579
Content of postnatal care:													
BCG vaccine given during postnatal period	7,767	8,576	7,706	9,972	9,594	8,989	11,058	8,966	8,710	6,941	**4,441**	6,422	99,142
Polio vaccine given at birth	5,256	6,571	5,589	5,916	7,151	6,803	7,506	6,483	6,588	6,093	7,143	7,297	78,396
Exclusive breastfeeding, 0–6 months	1,724	2,107	1,789	1,653	1,453	1,624	**3,103**	1,864	1,710	1,911	2,729	2,131	23,798

Notes:

TT = tetanus toxoid; IPT = intermittent preventive treatment in pregnancy for malaria; BCG = Bacillus Calmette-Guérin. Monthly values in **bold** indicate a moderate outlier between 2 and 3 standard deviations from the mean. Monthly values in ***italic and bold*** indicate an extreme outlier more than 3 standard deviations from the mean.

### Consistency between related data reported in DHIS2

When reviewing the extent to which data make sense with respect to each other (internal consistency between related indicators), the World Health Organization guidance recommended that pairs of data elements that we expect to be equal in value fall within ±10% of each other. For example, for Gombe State, it was expected that the number of facility births would equal the sum of live births and still births. Internal consistency between related data can also be examined by comparing the number of unique women who have accessed the facility for services (e.g., first antenatal care visits or facility deliveries) to the number of women who have received an individual service. If we find that not every woman has received the expected service, this could represent low service uptake or under-reporting. For example, the number of women tested for anemia could be compared to the number of first antenatal care visits; the use of partograph during delivery could be compared to the number of facility deliveries.

**Fig 3:**
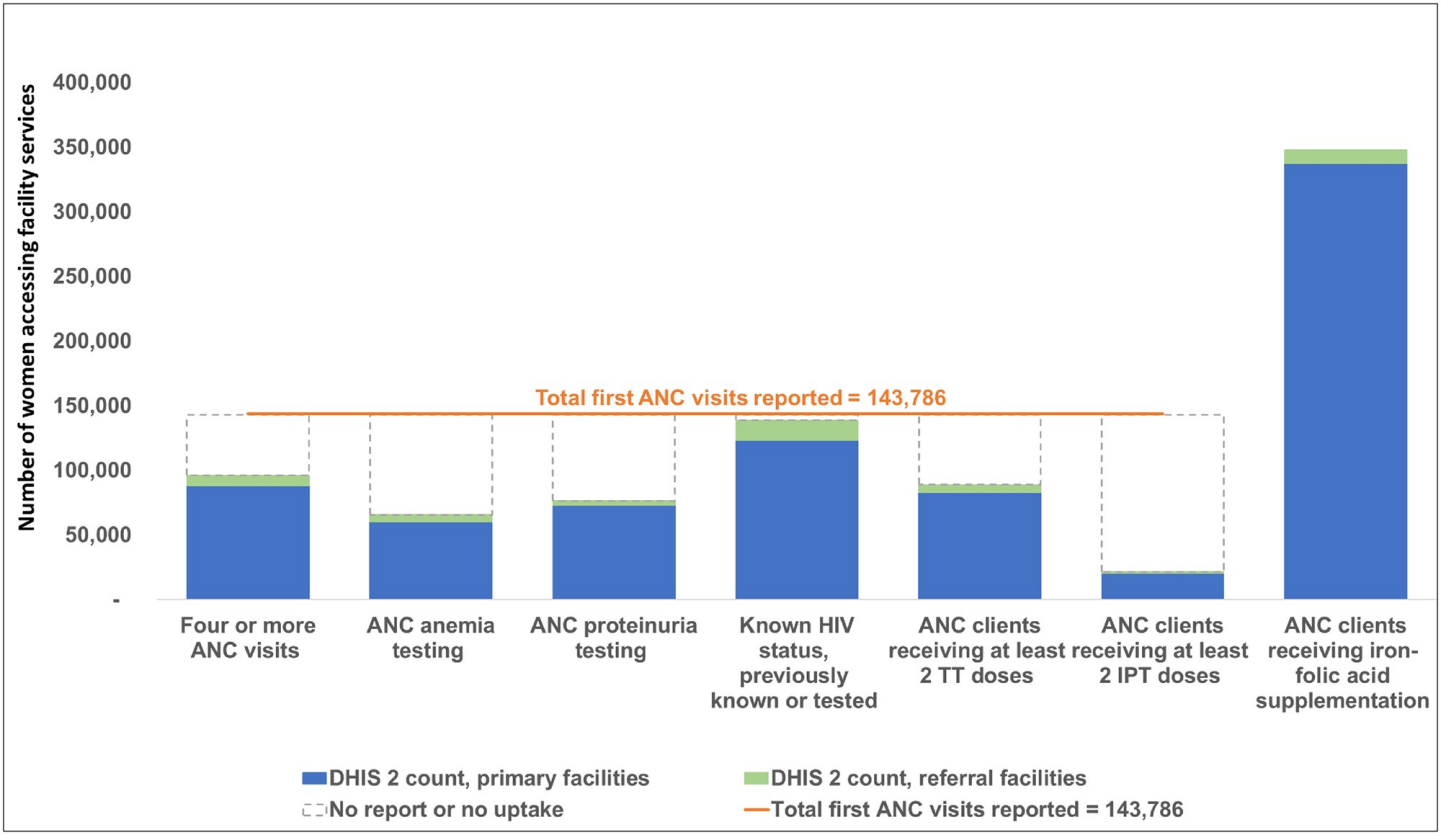
Consistency between related indicators: Facility-reported indicators for antenatal care in Gombe State, Nigeria, July 2016-June 2017, for 471 primary facilities and 26 referral facilities.

**Fig 4:**
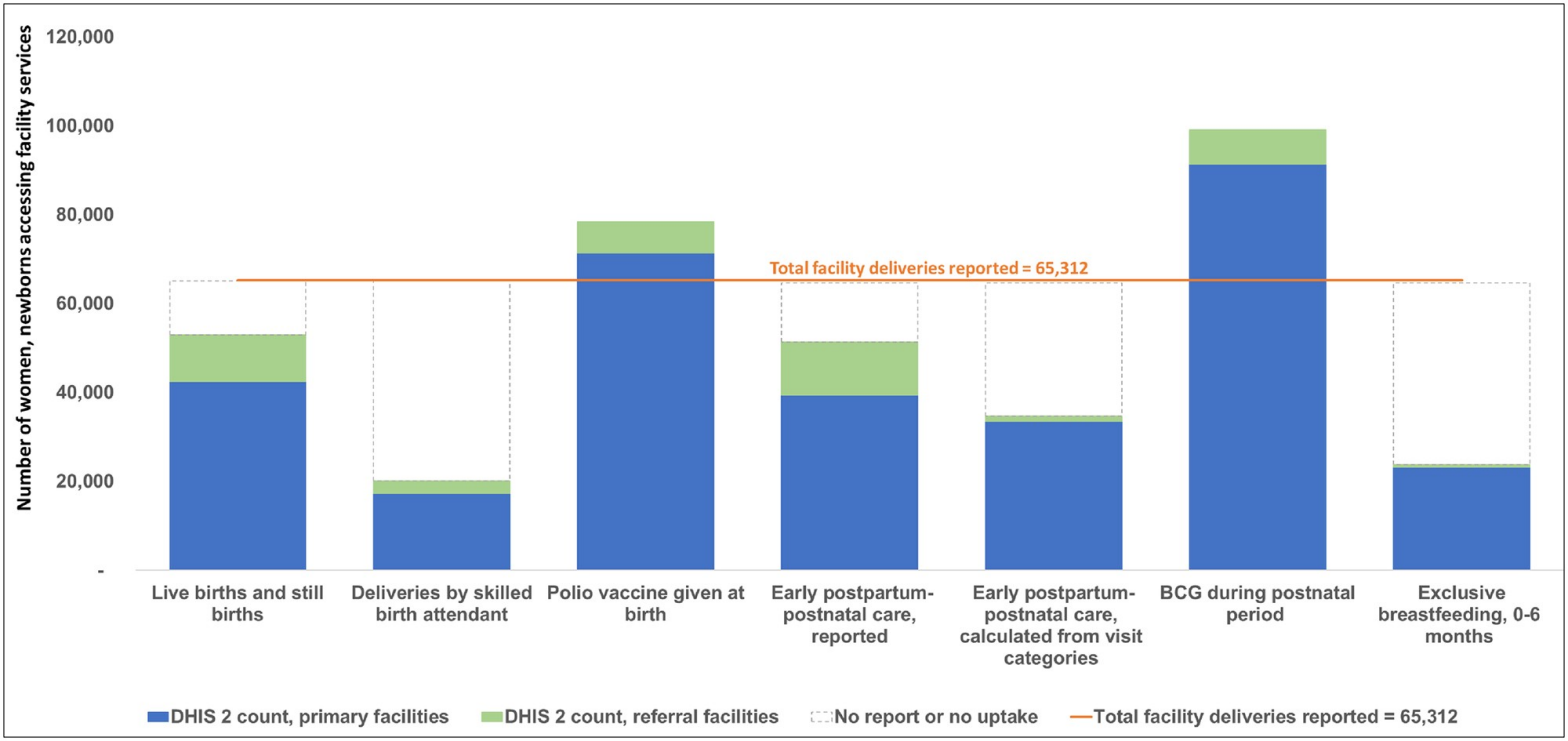
Consistency between related indicators: Facility-reported indicators for labor and delivery services in Gombe State, Nigeria, July 2016-June 2017, for 460 primary facilities and 26 referral facilities.

For Gombe State, related indicators that should have equal values did not meet the World Health Organization guidance: (i) the total number of deliveries (n = 65,312) did not equal the sum of live births and still births (n = 52,943) and (ii) the total reported number of women and newborns receiving early postpartum-postnatal care (n = 51,382) did not equal the sum of the visit categories (n = 34,686). (Fig 3 for antenatal and postnatal services, Fig 4 for labor and delivery services) Also in Figs 3 and 4, across all facilities, none of the priority data elements compared demonstrated the expected numerical relationship. For example, of the 143,786 first antenatal care visits, expected services provided during this first visit for anemia testing and proteinuria testing were reported for 65,732 women and 76,555 women, respectively. Primary facilities reported lower than expected numbers for 10 of the priority data elements which could be due to either low service uptake or under-reporting.

### Consistency between original facility registers and reported data in DHIS2

When assessing the extent to which data match across sources (consistency of original facility registers and reported data in DHIS2), the World Health Organization guidance defined the data to be consistent when the reported value (e.g., in DHIS2) was within ±10% of the facility register’s value. This review of consistency, in part, reflected the capacity to tally and report service statistics as intended.

**Fig 5:**
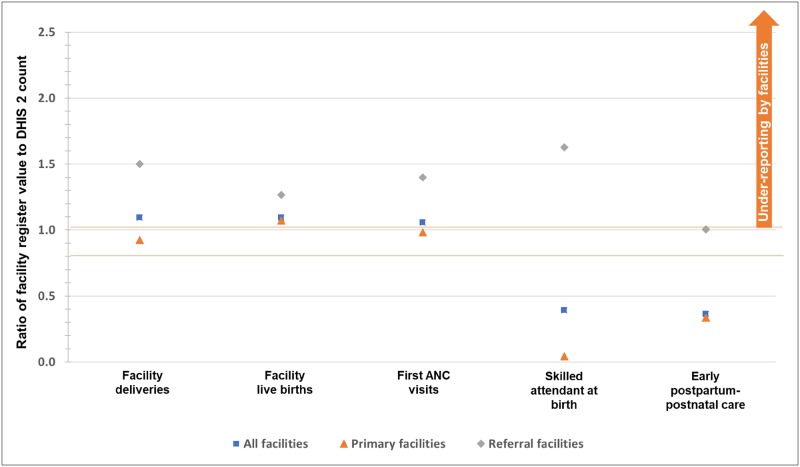
Consistency of data between original facility registers and reported data in DHIS2, January-June 2017. Notes: According to WHO guidance, ratios <0.9 or >1.1 indicate that reported data in DHIS2 were inconsistent with data extracted from the original facility register. For the 97 primary facilities where facility surveys and data extraction took place, five facilities offering antenatal and postnatal care services and seven facilities offering labor and delivery services were excluded as the facility registers were unavailable at the time of the survey.

For the five data elements compared (Fig 5), reported data in DHIS2 consistently agreed with the original facility registers for the priority indicators’ three main denominators: first antenatal care visits, facility deliveries, and live births. In general, facilities submitted higher numbers to DHIS2 (over-reported) compared to their original facility registers by 50%-60% for deliveries by skilled birth attendants and early postpartum-postnatal care.

By facility type, the frequency and magnitude of under-reporting was greater for referral facilities. While referral facilities’ data in DHIS2 consistently agreed with original facility records for early postpartum-postnatal care, referral facilities under-reported by more than 10% for the main denominators first antenatal care visits, facility deliveries, and live births and under-reported by more than 50% for deliveries by a skilled birth attendant. For primary facilities, data in DHIS2 consistently agreed with original facility registers for the abovementioned main denominators, but over-reported by more than 50% for deliveries by a skilled birth attendant and for early postpartum-postnatal care.

### External consistency between household surveys and reported data in DHIS2

When assessing the extent to which the data in DHIS2 are consistent with estimates from external data sources (external consistency), such as household surveys, the World Health Organization guidance recommended that the value of the routine data lie within the confidence limits or be within ±33% of the survey result. [23]

**Fig 6:**
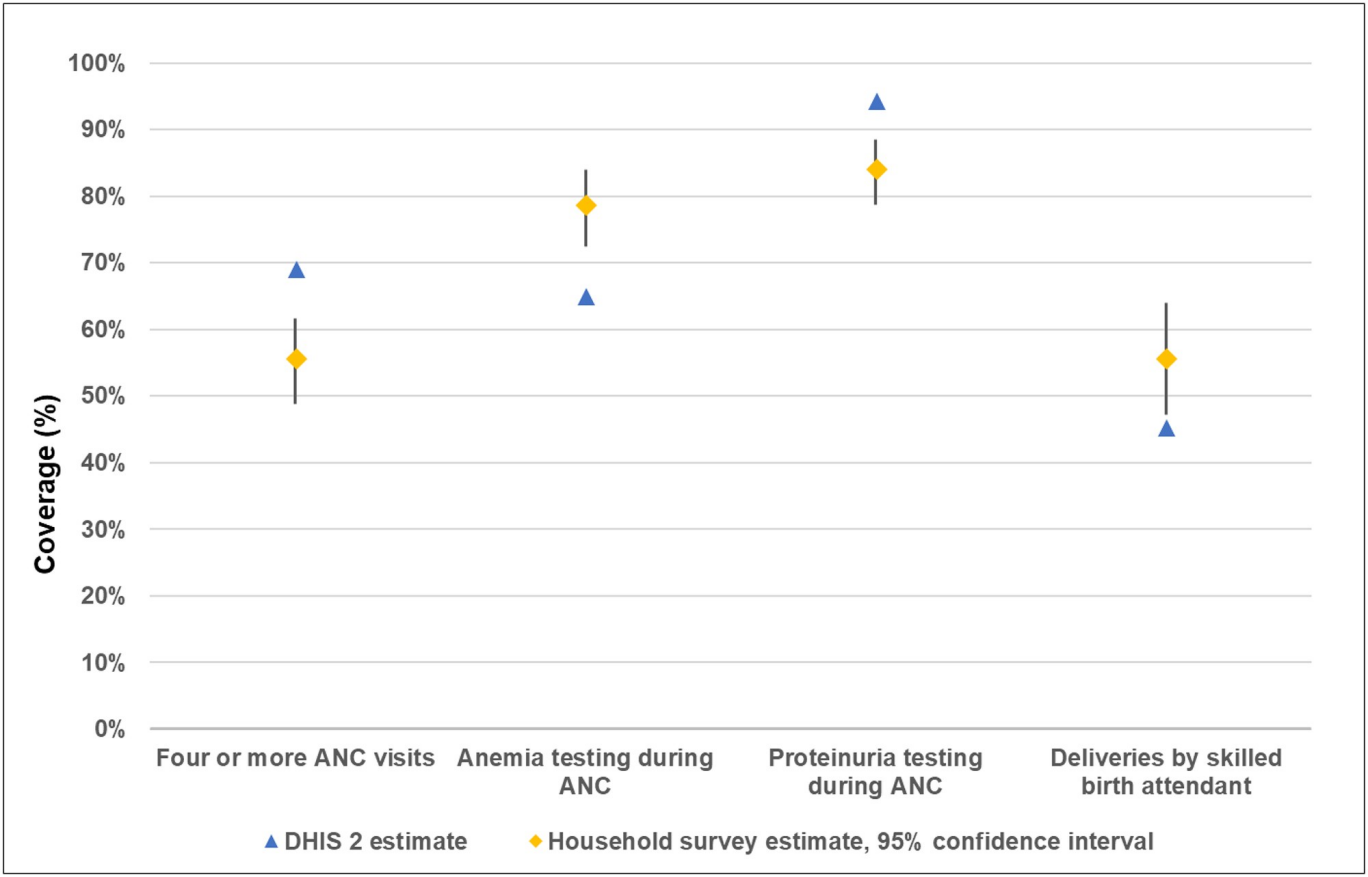
External consistency of priority MNH indicators, comparing DHIS2 data for July 2016-June 2017 with matched facility-clusters of a household survey in Gombe State, Nigeria (n = 79 facilities). Notes: ANC = antenatal care. Household survey denominator for (i) four or more ANC visits, (ii) anemia testing during ANC, and (iii) proteinuria testing during ANC: number of women who had received at least one ANC visit while pregnant during the one year prior to the survey (n = 377 women). Household survey denominator for deliveries by skilled birth attendant: number of women who had given birth in a facility during the one year prior to the survey (n = 588 women).

Fig 6 presents a comparison of the data reported in DHIS2 to the estimates from household surveys for four data elements. Comparing women who had visited a facility at least once during their pregnancy in 79 matching facilities and catchment areas, the routine data in DHIS2 did not fall within the confidence limits nor within ±33% of the survey results for the three antenatal care services reviewed: four or more antenatal care visits, women receiving a screening test for anemia, and women receiving a screening test for proteinuria. Comparing women who had delivered in a facility, the routine data in DHIS2 did not fall within the confidence limits nor within ±33% of the survey results for deliveries by a skilled birth attendant. Further, no pattern emerged in which the routine data in DHIS2 consistently overestimated or underestimated the results from the household survey.

## Discussion

We assessed the quality of routine data in DHIS2 to monitor priority maternal and newborn health indicators in Gombe State, north-eastern Nigeria. Of 14 facility-based indicators reflecting services that every woman and her newborn should receive, data elements to estimate 12 priority indicators were included in Gombe State’s DHIS2. However, similar to other studies assessing routine data, the routine maternal and newborn health data in DHIS2 for Gombe State did not meet all defined criteria for sufficient quality. [30–48]

During the reference year July 2016-June 2017, the data in DHIS2 did not regularly reflect what was in the facilities’ service registers, were incomplete, and exhibited inconsistencies over time, between related indicators, and with an external data source. Nevertheless, the data quality metrics assessed were not equally poor across all priority indicators. This variability suggests high quality routine data is achievable.[49] Data were of better quality when aligned with Gombe’s health program priorities, particularly for older health programs; there were also differences in data quality by indicator type.

Contact indicators, which reflect attendance at a facility, had the highest overall data quality among the priority indicators: first antenatal care visits, four or more antenatal care visits, facility deliveries, live births, deliveries by a skilled birth attendant, and early postpartum-postnatal care. [29, 50] These are well-defined events to document, which may ease the tallying and reporting of these data. In particular, the main denominators—first antenatal care visits, facility births, and live births—had the highest completeness of indicator data rates in our study, were more consistent over time, lacked extreme outliers, and demonstrated the greatest level of agreement between facility registers and DHIS2 data. Further, these have been key denominators for local program planning because they track the number of women accessing antenatal or postnatal care and labor and delivery services at health facilities. These data have been prioritized for monitoring progress in previous global initiatives including the Millennium Development Goals and Countdown to 2015. [51, 52] Four or more antenatal care visits and deliveries by a skilled birth attendant are also long-standing priority indicators for these initiatives and had the same data quality characteristics as the three denominators above. However, the lower overall data quality for deliveries by a skilled birth attendant may in part be reflective of the Gombe context where the majority of facility deliveries in primary facilities are managed by community health extension workers, rather than more highly trained nurses or doctors, following the recent political instability there.[53] Finally, early postpartum-postnatal care, an acknowledged “neglected period for the provision of quality care” had the lowest data quality metrics within this type of indicator. [54]

Indicators related to the provision of a commodity or vaccination that every woman or newborn should have received had the next highest level of overall data quality. While these indicators’ overall data quality was not as consistent as the contact indicators, they had relatively high completeness, relatively low inconsistencies over time and between related indicators. While reporting for these indicators may reflect the ease of accounting for a dispensed commodity, most commodities tracked by these indicators have been a part of Nigeria’s routine immunization program where completeness of indicator data and agreement across data sources have been emphasized.[55]

The last type of indicator reviewed is related to screening or testing pregnant women for anemia, proteinuria, or HIV. These had much lower completeness of indicator data rates, lower consistency between related indicators, and more outliers. These indicators reflect a more complex encounter between the client and health care provider and have been gaining attention as the maternal and neonatal health program priorities expand to include the content of care. [56, 57] They also tended to exhibit less consistency over time, possibly reflecting the increased attention. [58]

The configuration and use of DHIS2 in Gombe State underscores government commitment to using data to improve service delivery and health outcomes. As this study was reviewed from a program monitoring lens, we did not examine all facility-level characteristics and factors associated with data quality, but outline below actions at multiple levels that could improve data quality.

### Maximize the use of existing data

Data for two priority indicators of life-saving care are already captured at facility level, but are not included in monthly monitoring reports: women receiving oxytocin to prevent post-partum hemorrhage and newborns receiving essential newborn care. Monitoring these two indicators would align with recent efforts to focus on the content of care received at critical timepoints during labor and delivery. [59, 60]

### Rationalize data flow

Comparing facility registers to data entered in DHIS2, referred elsewhere as “accuracy”, highlighted differences by facility type. Both primary and referral facilities were affected by challenges in data flow. At the primary level, client antenatal “treatment” cards were often kept at the facility, and data were later transferred to a register, which was the primary data source for reporting. If the data from treatment cards had not been transferred to the register when the monthly report was prepared, data was taken directly from the cards into the monthly report, resulting in apparent over-reporting. At referral facilities, the physical task of gathering data from “treatment” cards and facility registers dispersed across the hospital grounds was a challenge. The person filling in the summary form was relatively far from services provided and relied on possibly incomplete or unavailable registers, resulting in under-reporting.

Our study suggested that facility staff could strengthen accuracy and completeness of documentation by ensuring that the most complete data source, whether it is the client’s antenatal treatment card or the service register, be the primary source for tallying and summarizing the services provided in the facility’s monthly report. At the state and national-levels, another action could be to review the role that the client cards play in the data flow, given that they remain in the facility as a medical record; a simple job aid could be developed to help tally across the treatment cards, rather than intensive data transcription to service registers that may no longer be fit for purpose. A cohort register, based on month of first antenatal visit date, could be developed to combine the longitudinal information needs of a treatment card with the tallying and summarization needs of the register; however, this type of tool development may not be realistic in the near-term.

### Routinize data quality reviews and feedback at all levels of the health system

Data quality review, feedback, and supervision are essential to optimize routine data for monitoring. [33, 35, 41, 43, 44, 46, 61–69] Studies specifically considering technology-based innovations, including DHIS2, noted that while innovations can make reviews of completeness and internal consistency more efficient, feedback and supervision remain essential to achieving and maintaining improvements in data quality. [33, 49, 66, 70] At the facility level, staff responsible for reporting should review the monthly reports for completeness and internal consistency, ensuring that related data elements have the expected numerical relationships, before submitting the report to the district level. This provides an important opportunity to review relationships between the number of clients and the services/commodities received to understand gaps in service uptake or gaps in data quality related to data capture or reporting; feedback can be provided to staff and supervised to address any gaps identified. At the district level, health management teams could take on these same practices and additionally structure their review and feedback to regularly allow for facilities to confront their own data and for comparison with neighboring or similar facilities in the context of where data quality metrics for completeness, timeliness, and internal consistency could be improved. [41, 43, 46, 62–64, 66, 67, 69, 71–73]

### Optimize and maintain DHIS2

Many global initiatives are looking to the DHIS2 platform to promote better quality data and improve access for monitoring at all health management levels. DHIS2’s platform allows Governments to develop a responsive information system. [33, 74] Based on our study, it is difficult to determine to what extent those features have been used to control data quality. For example, at the time of this review, DHIS2 for Gombe contained inactive facilities and administrative units, duplicate entries for active facilities and data elements, and did not distinguish between missing data and true zero values. [75, 76] These required additional preparation for our analyses, suggesting that comprehensive data quality reviews could not take place in DHIS2 in its current form. An investment in DHIS2 should include ongoing reviews of its content to promote data quality and fitness for purpose.[11, 33, 35, 70, 74]

There were limitations to this study. Similar to other assessments, we did not validate the data through direct clinical observations [41, 43–45, 49, 64, 77] nor did we compare the paper-based monthly summary reports to their electronic versions in DHIS2. [32, 34, 41, 44, 61, 78] For the assessment of consistency, the facility-level and household-level surveys used in this study could not be considered a gold standard, but we did consider them to be relevant references for reviewing the consistency of routine facility-based data in DHIS2. Understanding consistency between multiple data sources is a perennial problem for health managers who frequently have to make sense of different estimates. The surveys were conducted similarly to the Demographic and Health Surveys and Multiple Indicator Cluster Surveys at the household-level, and the Service and Readiness Assessment at the facility-level, where estimates of priority maternal and newborn coverage and service delivery indicators have been obtained.[28, 79, 80] Despite close attention to quality control, these surveys might still be susceptible to errors in data recording, including incorrectly tallying the number of events in the original facility registers for comparison with data in DHIS2. Further, for some maternal and newborn health events, household survey measures may not provide a valid representation of care provided in health facilities.[81] Acknowledging these short-comings highlights the importance of working to improve the utility of routine data sources. We did not review rare events or outcomes such as deaths or complications and extra care for women and their newborns, as our primary interest was in the contribution of DHIS2 data to routine program monitoring. Lastly, this study reviewed the quality of routine data for maternal and neonatal health and may not be representative of indicators for the planning and service provision for other health programs.

Our study adds new evidence showing the potential of data in DHIS2 for local, real-time monitoring of maternal and newborn health services. While the quality of data in DHIS2 could be strengthened, the data quality metrics for priority indicators were not universally nor equally poor. Coordinated action at multiple levels of the health system is needed to maximize reporting of existing data; rationalize data flow; routinize data quality review, feedback and supervision; and ensure the ongoing maintenance of DHIS2.
